# Epidemiologic Characteristics of Mpox among People Experiencing Homelessness, Los Angeles County, California, USA, 2022

**DOI:** 10.3201/eid2906.230021

**Published:** 2023-06

**Authors:** Hannah K. Brosnan, Karen W. Yeh, Padma S. Jones, Sohum Gokhale, Dalia Regos-Stewart, Hang Tran, Kathleen Poortinga, Phoebe Danza, Rebecca Fisher, Lauren E. Finn, Chelsea Foo, Alicia H. Chang

**Affiliations:** Los Angeles County Department of Public Health, Los Angeles, California, USA

**Keywords:** Mpox, homelessness, surveillance, viruses, sexually transmitted infections, underlying health conditions, California, United States, monkeypox virus

## Abstract

In Los Angeles County, California, USA, public health surveillance identified 118 mpox cases among persons experiencing homelessness (PEH) during July–September 2022. Age and sex were similar for mpox case-patients among PEH and in the general population. Seventy-one (60%) PEH mpox case-patients were living with HIV, 35 (49%) of them virally suppressed. Hospitalization was required for 21% of case-patients because of severe disease. Sexual contact was likely the primary mode of transmission; 84% of patients reported sexual contact <3 weeks before symptom onset. PEH case-patients lived in shelters, encampments, cars, or on the street, or stayed briefly with friends or family (couch surfed). Some case-patients stayed at multiple locations during the 3-week incubation period. Public health follow-up and contact tracing detected no secondary mpox cases among PEH in congregate shelters or encampments. Equitable efforts should continue to identify, treat, and prevent mpox among PEH, who often experience severe disease.

Since May 2022, laboratory-confirmed cases of mpox have been reported across nonendemic countries including the United States, mostly among men who have sex with men (*1**,**2*). Persons experiencing homelessness (PEH) are disproportionately affected by infectious diseases compared with the general population because of several factors, including close living quarters in shelters and encampments; lack of consistent access to hygiene facilities when living on the streets; less access to healthcare services; and coexisting medical, mental, and substance use disorders that may increase susceptibility or pose barriers to prevention and treatment (*3**–**6*). Minimal literature exists on the characteristics and epidemiology of mpox among PEH. Los Angeles County, California, USA has a large, heterogeneous PEH population, estimated at 69,144 persons experiencing sheltered and unsheltered homelessness; 10% identify as gay, lesbian, bisexual, or questioning (*7*,*8*). Because widespread transmission of mpox among PEH became a concern at the outset of the local outbreak, the Los Angeles County Department of Public Health (LACDPH) initiated this study to review demographics, discern patterns of transmission, and identify risk factors unique to PEH to better understand the effect of mpox among that vulnerable population and determine the need for changes to existing surveillance and mitigation strategies. This study was evaluated by an LACDPH internal review board, which determined it meets criteria for not being human subject research and review was not needed. 

## Methods

In accordance with Centers for Disease Control and Prevention (CDC) emergency mpox response guidelines, LACDPH implemented mpox case surveillance and investigation beginning in May 2022 (*9*). We handled both probable and confirmed cases reported to LACDPH equally as actual mpox cases (*10*). In addition to mandatory laboratory reporting of positive mpox and orthopox virus test results, LACDPH required healthcare providers to report all mpox or orthopoxvirus infections, hospitalizations, and deaths within 1 working day after identification using an online form (*11*,*12*). Medical provider forms were used to collect demographic, clinical, and epidemiologic information, including preinfection residential situations (Appendix Table 1). Providers had the option to send photographs and additional medical records to LACDPH to complement the mandatory report form. We combined race and ethnicity data from all mpox case reports. Classification options were mutually exclusive and consisted of black or African American, Latinx/Hispanic, white, and other. All case-patients reporting Latinx/Hispanic ethnicity were grouped into that category regardless of any racial identification. Case-patients identifying as American Indian/Alaska Native, Asian, multirace, Native Hawaiian/Pacific Islander, or any other unspecified category, were grouped under the category other because <5 case-patients indicated each of those options. 

LACDPH matched mpox cases to HIV cases in the electronic HIV registry (eHARS) to obtain co-infection, viral suppression, and CD4 counts. We categorized mpox cases as virally suppressed if the most recent HIV viral load on record was <200 copies/mL and performed <12 months before mpox diagnosis. We used most recent CD4 counts after mpox diagnosis to categorize HIV/mpox co-infections by level of immune suppression. 

Trained investigators conducted structured interviews with all mpox case-patients reachable by phone or in person to collect additional risk factor data, assist with isolation housing and treatment, and initiate contact tracing; 3 phone calls, 3 texts, and 2 home visits were attempted for each case. Interview data included sexual orientation, symptoms and clinical history, employment, housing status and locations (Appendix Table 2), sexual history during the 3-week mpox incubation period before symptom onset, and other behavioral characteristics. The interviewer also asked mpox case-patients to name and provide phone numbers and addresses for all their intimate contacts. After the initial interview, we contacted mpox case-patients weekly until symptoms resolved and also gathered follow-up data on hospitalizations and treatments. Potential mpox contacts for whom we had information were called, texted, or visited by LACDPH staff for follow-up to review symptoms or arrange for mpox testing or vaccination. 

LACDPH verified housing status for mpox case-patients for whom homelessness was noted in the mandatory healthcare provider report forms or who answered affirmatively to experiencing homelessness in the interview. The purpose of verifying housing was to confirm or amend homelessness status according to Department of Housing and Urban Development (HUD) and CDC definitions (*13*,*14*) during the 3 weeks before symptom onset. Verification methods included requesting and reviewing medical records from hospitals or clinics, and cross-checking against records from existing LACDPH communicable disease databases, other Los Angeles County department databases, and the local Homeless Management Information System, the data system required by HUD for providers receiving federal funds for the administration of homeless services (*15*). The Homeless Management Information System contains cumulative profiles and service records of persons who have entered emergency, transitional, or permanent shelter or who have received street outreach services for care and case management. 

We included in this report mpox cases diagnosed among PEH during July 16–September 22, 2022. After verifying homelessness status, we categorized PEH case-patients by primary residential situation on the basis of where they spent the highest number of nights during the 3-week incubation period; we also recorded, categorized, and referred for public health follow-up additional locations where case-patients slept during the 3-week incubation period. Location categories were sheltered–congregate (emergency, transitional, and domestic violence shelters, and recuperative care centers); sheltered–other (noncongregate temporary housing such as hotels, motels, or couch surfing [staying briefly with friends or family] in private homes); unsheltered–encampment (living with others in places or structures not meant for human habitation, such as parks, streets, or vehicles); unsheltered–other (living alone in places or structures not meant for human habitation, such as parks, streets, or vehicles); and unknown. 

We referred facility addresses identified during the interview and verification processes to field public health nurses who worked with facility staff to set up any necessary activities for outreach, education, symptom surveillance, clinical evaluation and testing, and vaccination of staff and residents. Each site was monitored for >3 weeks after an infectious PEH mpox case-patient was moved to dedicated isolation housing. If there were additional symptomatic persons reported, each site was monitored further until negative results were reported from mpox or orthopox testing. We cross-referenced locations for all mpox cases among PEH and among the general population when sufficient address information was available. We performed all analyses using SAS version 9.4 (SAS institute, https://www.sas.com). 

## Results 

During July 16–September 22, 2022, a total of 118 mpox cases among PEH and 1,805 total mpox cases were reported in Los Angeles County. PEH cases peaked at 26 during the week of August 6, 2022, whereas non-PEH cases peaked at 271 the week of July 30 (Figure). Most (62/118, 53%) PEH mpox case-patients were 30–39 years of age (Table 1). By gender identity, 108 (92%) identified as men, 5 (4%) as women, and 5 (4%) as other (includes transgender female, transgender male, or another gender identity). By race/ethnicity, 49 (42%) were categorized as Latinx/Hispanic, 37 (31%) black or African American, 18 (15%) white, and 7 (6%) other (American Indian/Alaska Native, Asian, multirace, or Native Hawaiian/Pacific Islander); race/ethnicity were unknown for 7 (6%). By sexual orientation, 64 (54%) identified as gay or lesbian, 21 (18%) as bisexual or by another term, and 14 (12%) as straight or heterosexual; 5 (4%) preferred not to state, and sexual orientation was unknown for 14 (12%). 

**Figure:**
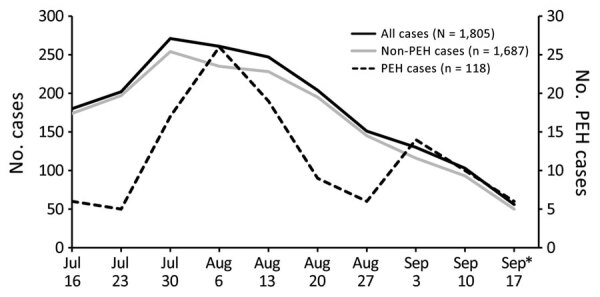
Mpox cases by week among persons experiencing homelessness, Los Angeles County, California, USA, July 16–September 22, 2022. Scales for the y-axes differ substantially to underscore patterns but do not permit direct comparisons.

**Table 1:** Characteristics of mpox case-patients experiencing homelessness, Los Angeles County, California, USA, July 16–September 22, 2022*

Characteristic	No. (%)
Total	118 (100)
Age, y	
0–17	0 (0)
18–29	20 (17)
30–39	62 (53)
40–49	27 (23)
>50	9 (8)
Unknown	0 (0)
Gender identity	
M	108 (92)
F	5 (4)
Other†	5 (4)
Race/ethnicity‡	
Black/African American	37 (31)
Hispanic	49 (42)
White	18 (15)
Other: American Indian/Alaska Native, Asian, Native Hawaiian/Pacific Islander, multirace, other race	7 (6)
Unknown	7 (6)
Sexual orientation	
Gay or lesbian	64 (54)
Straight or heterosexual	14 (12)
Bisexual or another term	21 (18)
Prefer not to state	5 (4)
Unknown	14 (12)
HIV status§	
Positive	71 (60)
No match to HIV registry	47 (40)
Viral suppression among PEH with HIV,¶ n = 71	
Y	35 (49)
N	36 (51)
CD4 count, cells/mm^2^, among PEH with HIV and prior CD4,# n = 70	
<200	10 (14)
>200	60 (85)
Primary living situation**	
Sheltered–congregate	8 (7)
Sheltered–other	55 (47)
Unsheltered–encampment	12 (10)
Unsheltered–other	37 (31)
Unknown	6 (5)

*PEH, people experiencing homelessness. †Transgender male, transgender female, gender nonbinary, gender nonconforming, other gender. ‡Race categories were based on self-reports from laboratory reports or interviews. Race categories with <5 respondents were combined as other (American Indian/Alaska Native, Asian, Native Hawaiian/Pacific Islander, multirace, other race). §HIV status according to case match to HIV registry. ¶Most recent HIV viral load <200 copies/mL occurring within the prior 12 months. #Most recent CD4 count. **Primary living situation at time of exposure. Sheltered–congregate: specifically in congregate sheltered settings such as homeless or transitional shelters, domestic violence shelters, recuperative care centers; sheltered–other: noncongregate temporary housing such as hotels/motels used for emergency housing, or couch surfing in private homes; unsheltered–encampment: sharing residence with groups of other persons in places or structures not meant for human habitation; unsheltered–other: residing in places or structures not meant for human habitation such as parks, streets, or vehicles.

HIV prevalence among PEH mpox case-patients was 60% (N = 71), among whom 36 (51%) had unsuppressed viral loads and 10 (14%) had a CD4 count <200 copies/mL. Clinical severity of mpox required hospitalization for 25 (21%) PEH, 19 of whom were HIV positive. A total of 42 (35%) PEH mpox case-patients received treatment with tecovirimat. 

Among the 118 PEH mpox case-patients, public health staff were able to locate and interview 101 (86%) (Table 2). Of those interviewed, 21 (21%) reported exposure to a known or symptomatic mpox case-patient; none named exposure sources or provided additional details. When those 21 were cross-checked against records from all mpox cases, only 1 was named as a contact in a mpox case among the general population. 

**Table 2:** Self-reported behavioral characteristics among mpox case-patients experiencing homelessness interviewed in Los Angeles County, California, USA, July 16–September 22, 2022

Characteristic	No. (%)
Total interviewed*	101 (86)
Reported exposure to monkeypox case	
Y	21 (21)
N	45 (45)
Don't know	34 (34
Did not answer	1 (1)
Attended large public or private event	
Y	24 (24)
N	75 (74)
Did not answer	2 (2)
Traveled	
Y	5 (5)
N	94 (93)
Did not answer	2 (2)
Had sexual contact with >1 partners	
Y	74 (73)
N	23 (23)
Did not answer	4 (4)
No. of partners, n = 74	
Unknown	2 (3)
1	30 (40)
2–5	31 (42)
>6	11 (15)
Venues for meeting sex partners,† n = 74	
Online apps	34 (46)
Social event, bathhouse, sex club	5 (7)
Other	20 (27)
All other venues	17 (23)
Not applicable, e.g., long-term partner	11 (15)
Participated in group sex, n = 74	
Y	9 (12)
N	65 (88)
Gave or received drugs/money/favors/food/housing for sex, n = 74	
Y	11 (15)
N	63 (85)
Signs and symptoms†	
Rash, including lesions or skin bumps	96 (95)
Malaise: general feeling of illness/weakness	63 (62)
Fever	62 (61)
Enlarged lymph nodes	54 (53)
Headache	51 (51)
Myalgia	49 (49)
Chills	47 (47)
Pruritis	45 (45)
Back pain	31 (31)
Vomiting or nausea	24 (24)
Rectal pain	23 (23)
Cough	22 (22)
Abdominal pain	21 (21)
Runny nose	17 (17)
Pus or blood on stools	14 (14)
Rectal bleeding	11 (11)
Eye lesions	7 (7)
Conjunctivitis	5 (5)
Tenesmus	5 (5)

*Among 118 mpox case-patients experiencing homelessness. †Not mutually exclusive categories.

A total of 24 (24%) PEH mpox case-patients reported attending a large event <3 weeks before symptom onset. Seventy-four (73%) reported sexual contact; 23 (23%) denied sexual contact. Among those who were sexually active, 30 (41%) reported 1 sexual partner, 31 (42%) 2–5 partners, and 11 (15%) >6 partners. Nearly half (34/74; 46%) of sexually active PEH mpox case-patients reported meeting partners through mobile phone applications; others reported meeting through bath houses, sex clubs, or social events. Nine (12%) respondents reported engaging in group sex; 11 (15%) reported participating in transactional sex, defined as exchanging sex for money, drugs, food, housing, or other unspecified favors. 

Among the 23 PEH mpox case-patients who denied sexual contact during the structured interview with public health investigators, 11 (48%) reported sexual contact to their healthcare providers as documented in notes within the mandatory reporting forms or medical records that were submitted to LACDPH. One of those 11 case-patients reported having been sexually assaulted to public health staff outside of the structured interview. Among the 12 case-patients with no report of sexual activity from interviews or records, 3 reported other possible sources of mpox transmission (1 self-reported trying on unwashed found clothing; 1 reported sharing food, utensils, dishes, bathrooms, and razor blades; and 1 reported staying at a shelter), although investigators were unable to confirm those sources. The other 9 mpox case-patients with no report of sexual activity reported no other possible sources of transmission. 

Using data from healthcare provider reports and mpox case interviews, LACDPH was able to determine the primary residential situation in the 3 weeks before symptom onset for 112 (95%) PEH mpox case-patients; 55 (47%) were grouped in the sheltered–noncongregate category. Of these, 49 were couch surfing in private homes, 4 used temporary vouchers to stay in private rooms with bathrooms in hotels/motels, and 2 stayed in private rooms with bathrooms in hotels/motels used specifically for emergency housing. Of 37 (31%) PEH grouped as unsheltered–other, 18 were living outdoors but not associated with an encampment, 10 were unsheltered with details unknown, and 9 were living in vehicles. We grouped 12 (10%) in the unsheltered–encampment category, 8 (7%) as sheltered–congregate, and 6 (5%) as unknown (Table 1). Twenty-nine (25%) PEH mpox case-patients reported spending nights at >1 location within the 3-week incubation timeframe before onset of symptoms, among whom 14 spent most nights couch surfing (7 moved around from private home to private home), 5 spent some nights outdoors or in a vehicle, 4 spent time in a commercial hotel, 1 in an emergency shelter, 1 incarcerated, and 1 in a non-PEH setting; 1 additional location was unknown. No mpox case-patient was identified as sharing the same encampment or address with another case-patient. 

Among the 21 PEH mpox case-patients who reported exposure to a person with known mpox or mpox symptoms, 10 couch surfed, 6 lived in encampments, 2 lived in emergency shelters, and 3 lived alone on the streets. One of the 21 case-patients who reported exposure to a known mpox case-patient reported exchanging sex for services. Of the 11 PEH mpox case-patients who reported exchanging sex for services, 5 were sheltered (3 couch surfing, 1 living in a group home, and 1 in a shelter) and 6 were unsheltered (1 in an encampment, 2 in vehicles, and 2 alone on the streets). There were no additional details for 1 of the unsheltered PEH mpox case-patients who exchanged sex for services. 

## Discussion

In this large descriptive series of mpox cases among PEH, mpox case-patients were proportionally similar by age and race to the underlying PEH population in Los Angeles County but disproportionally by sex (higher male proportion) (*7*,*8*). Our finding of a high proportion of male than female PEH mpox case-patients is similar among the general population (*16*). No mpox cases were identified among minors experiencing homelessness. HIV prevalence was higher among PEH mpox case-patients (60%) than among overall mpox case-patients reported from Los Angeles County and 7 other US jurisdictions (38%) (*17*). Positive referral bias might partially explain higher documented HIV prevalence; PEH with poorly controlled HIV might be more likely to seek care and receive a diagnosis because of more severe mpox illness. However, it is also possible that PEH with HIV are more susceptible because of discontinuous HIV care, disruptions in housing, and other risk factors, which might indicate higher actual prevalence. 

After acquiring mpox, PEH are more vulnerable to severe disease. CDC reported 23% of persons with severe mpox who received medical consultation services through direct requests from local jurisdictions were PEH (*18*). Among our cohort of 118 PEH mpox case-patients, disease was severe enough in 21% to require hospitalization, and consistent with CDC findings, those hospitalizations comprised 27% of all mpox hospitalizations in Los Angeles County (data not shown). Los Angeles County maintains dedicated isolation housing outside of clinics for PEH mpox cases, so those hospitalizations were not for the purpose of isolation or housing. Additional details on coexisting medical conditions other than HIV that may have contributed to disease severity were not available and remain gaps in the data. 

Of the 47% of mpox case-patients in sheltered–noncongregate settings, 89% were couch surfers, who are difficult to identify using traditional surveillance methods without dedicated questions delving into housing details. Couch surfers are not included in PEH population estimates from the point-in-time counts required by HUD in the Continuums of Care (*19*) and may rapidly cycle between private homes and streets to shelters (*20*). PEH who predominantly couch surf warrant further study to better understand their risk factors for communicable diseases and inform disease prevention strategies. 

Similar to the situation for mpox cases among the general population, the primary mode of transmission for PEH mpox cases appeared to be through sexual contact; 84% of PEH mpox case-patients reported this risk factor, 73% to an LACDPH interviewer and 11% to another healthcare provider. California lists mpox under the California Division of Occupational Safety and Health’s Aerosol Transmissible Diseases standards, which includes both aerosol-borne diseases and select diseases transmitted through droplets (*21*). This designation requires shelter employees to use more stringent protections, including wearing fit-tested N95 respirators when interacting with persons suspected of having or confirmed to have mpox infection. However, despite our initial concerns about respiratory transmission of mpox and potential spread through droplets or fomites in congregate settings, we found no evidence of any transmission within shelters to either PEH or staff. Masking requirements in response to COVID-19 in Los Angeles County during the 2022 mpox outbreak may have affected mpox transmission in congregate settings. However, the lack of transmission within shelters is consistent with anecdotal reports from other jurisdictions (San Francisco Department of Public Health, New York State Department of Health, pers. comm., email, July 26, 2022) and with a Cook County, Illinois, USA, report of an exposure in a correctional facility where investigation by symptom monitoring and serologic testing after a single mpox case in a jail resident found no secondary cases (*22*). Similarly, no transmission in encampments, considered congregate settings by Los Angeles County, was identified despite potential sharing of sleeping bags, clothes, and utensils in settings with poor access to cleaning and laundry services. One PEH mpox case-patient did report exposure without sexual contact through wearing found clothing. Additional research is needed to identify nonsexual transmission among PEH, especially among encampment residents where follow-up and contact tracing are challenging. Transmission among couch surfers appeared to follow patterns among the general population. Addresses provided by couch surfers did not match addresses for any other recorded mpox case, so it was difficult to fully assess transmission characteristics for couch surfers. 

Among limitations to this report, LACDPH surveillance data were limited by reliance on provider and laboratory reporting of positive test results. In a historically marginalized population that experiences multiple barriers to healthcare, it is probable that not all PEH with mpox symptoms received the necessary medical attention for diagnosis and treatment. A serosurvey conducted by CDC among 209 persons experiencing homelessness found 3 possible missed cases of mpox (*23*), suggesting a small, but present, negative case detection bias from mpox surveillance based on case reporting. This bias may also have affected LACDPH’s assessment of transmission within shelters and encampments, particularly because contact tracing is more challenging among PEH than among the general population. Symptomatic persons may have been afraid of the stigma of mpox or losing housing and not come forward despite receiving public health outreach, education, onsite testing, and vaccination in shelters and encampments, which had 1 reported mpox case among PEH. LACDPH field staff relied on self-reports and did not conduct physical exams or serology testing as part of onsite follow-up. 

In addition, case-patient information was self-reported through interviews, and LACDPH had minimal ability to confirm or verify responses. For example, among the 21 persons who reported contact with an mpox case-patient, no confirmation was possible because case-patients provided no contact names. Because sexual history can be a sensitive topic, mpox case-patients might have been hesitant to disclose information to a public health investigator who had no previous therapeutic relationship with the patient. The 11 persons who disclosed sexual encounters only to healthcare providers other than the interviewer, and the revelation outside of the interview by 1 PEH of having been sexually assaulted, suggests collection of incomplete risk factor data. In addition, this experience with collecting data from persons affected by a sexually transmitted disease reinforces the need for public health surveillance and interventions to be designed and implemented with sensitivity within a trauma-informed framework. 

Our findings illustrate the medical vulnerability of PEH, the heterogeneity of their living situations, and the importance of designing disease surveillance methods that capture the complex risk factors and exposures unique to this population. Questionnaires that include sensitive topics may be more successful when implemented after a therapeutic or other trust-based relationship has been established. Developers of public health interventions to prevent and control disease among PEH should consider how differences in living situations can affect disease transmission. Equitable public health efforts should continue to identify, treat, and prevent mpox cases among PEH, who often experience severe cases in part because of barriers to accessing healthcare. 

Appendix Additional information about mpox cases among people experiencing homelessness, Los Angeles County, California, USA, July 16–September 22, 2022.
