# Supplementary material for: Epidemiologic Characteristics of Mpox among People Experiencing Homelessness, Los Angeles County, California, USA, 2022
Source: Emerg Infect Dis. 2023 Jun;29(6):1109–16. doi: 10.3201/eid2906.230021 (PMC10202883; doi:10.3201/eid2906.230021)
Supplement: Appendix — Additional information about mpox cases among people experiencing homelessness, Los Angeles County, California, USA, July 16–September 22, 2022. [file 23-0021-Techapp-s1.pdf]

Article DOI: https://doi.org/10.3201/eid2906.230021
EID cannot ensure accessibility for supplementary materials supplied by authors. Readers who have difficulty accessing supplementary content should contact the authors for assistance.

Epidemiologic Characteristics of Mpox Infections among People Experiencing Homelessness, Los Angeles County, California, USA, 2022

Appendix

Appendix Table 1. Los Angeles County Department of Public Health Mpox Confidential Morbidity Report 2022

Question	Response Options
In the three weeks before illness onset and during illness, where did the patient reside (check all that apply)?	Private residence<br>Hotel<br>Shelter (homeless shelter, domestic violence shelter, etc.)<br>Unsheltered (street, encampment, etc.)<br>Couch surfing<br>Correctional facility<br>Nursing home/long-term healthcare facility<br>Residential care/assisted living facility<br>School/university dorm<br>Military base<br>Other
If selected Other: Please specify other location of residence	Free text

Appendix Table 2. Los Angeles County Department of Public Health Mpox Case Interview Form, 2022

Question	Response Options
Do you have a permanent address?	Yes/No
In the three weeks before your symptoms began, were you ever sleeping on the streets, in a shelter, tent, or encampment, or couch surfing?	Yes/No
If multiple shelters, please specify name and address for each:	Free text
If streets, tent, encampment, or couch surfing: Nearest cross streets/neighborhood/description of location:	Free text
If multiple location, please specify location for each:	Free text
In the last 3 weeks, have you lived or worked at a congregate living facility? (A congregate living facility is a living facility that offers living, sleeping, food, and other needs for residents)	Yes/No<br>Lived/Worked
In the three weeks before your symptoms began, were you ever sleeping on the streets, in a shelter, tent, or encampment, or couch surfing?	Yes/No
If shelter	Shelter name:<br>Shelter street address:<br>Shelter city:<br>Shelter state:<br>Shelter zip code:
If multiple shelters, please specify name and address for each:	Free text
If streets, tent, encampment, or couch surfing: Nearest cross streets/neighborhood/description of location:	Free text
If multiple locations, please specify location for each:	Free text
